# Impact of the COVID-19 Pandemic on the Oral Health Workforce: A Multicenter Study from the Southern Region of Brazil

**DOI:** 10.3390/ijerph20021301

**Published:** 2023-01-11

**Authors:** Cristine Maria Warmling, Rubens Spin-Neto, Luciana Zambillo Palma, Manoelito Ferreira Silva-Junior, Renata Goulart Castro, Mirelle Finkler, Márcia Helena Baldani, Fernando Valentim Bitencourt

**Affiliations:** 1 Graduate Program Teaching in Health, Federal University of Rio Grande do Sul, Porto Alegre 90010-150, RS, Brazil; 2 Department of Dentistry and Oral Health, Section for Oral Radiology, Aarhus University, 8000 Aarhus, Denmark; 3 Department of Health I, State University of Southwest Bahia (UESB), Jequie 45083-900, BA, Brazil; 4 Department of Dentistry, Federal University of Santa Catarina, Florianópolis 88040-900, SC, Brazil; 5 Department of Dentistry, State University of Ponta Grossa, Ponta Grossa 84010-330, PR, Brazil; 6 Department of Dentistry and Oral Health, Section for Periodontology, Aarhus University, 8000 Aarhus, Denmark; 7 Steno Diabetes Center Aarhus, 8200 Aarhus, Denmark

**Keywords:** COVID-19, dental education, health workforce, public health dentistry, SARS-CoV-2

## Abstract

The aim of this study was two-fold: (1) to describe the surveillance and biosafety measures adopted by dentists, dental hygienists, and dental assistants who worked in the Southern Region of Brazil and (2) to evaluate access to information in the context of the COVID-19 pandemic. This was a multicenter and cross-sectional design, using a self-applied and validated online questionnaire. The availability of health-care-related supplies and the adoption of biosafety measures recommended by the Technical Note of the National Health Surveillance Agency No. 04/2020 were analyzed. A total of 2560 Brazilian workers participated (75.8% dentists, 15.7% dental assistants and 8.5% dental hygienists), 52.7% from the public and 37.7% from the private sector. Approximately 70% of the individuals reported being away from work during the pandemic. The surveillance measures adopted with higher mean scores were the investigation of respiratory infection symptoms when scheduling appointments and the adoption of distancing in the waiting room. Of the biosafety measures to avoid aerosols, the procedures with lower compliance were those related to the use of intraoral radiographs and rubber dams. Moreover, the correct use of personal protective equipment at work seems to be related to self-perceived stress and anxiety. Worryingly, high access to information through non-governmental documents was observed. Permanent health education policies should reinforce safe practices and encourage workers to implement biosafety and surveillance measures in health services.

## 1. Introduction

The COVID-19 pandemic and its repercussions have overburdened health systems worldwide. In Brazil, approximately 75% of the Brazilian population depends exclusively on the Unified Health System (SUS), but political instabilities interfered with SUS’s organizational capacity to generate coordinated actions for pandemic control [1,2,3]. The magnitude of the spread of SARS-CoV-2 in the Brazilian national territory was broad and severely impacted the population [4]. Social inequalities were potentiated and became expressed through inequalities in the COVID-19 care networks [5]. The outcome of dealing with COVID-19 was catastrophic, and the country had the second-highest number of daily cases and deaths worldwide [6].

Expanding and strengthening the health workforce has become imperative in the face of the COVID-19 pandemic and its implications for the SUS. It is estimated that 2 million SUS workers assisted millions of contaminated individuals [7,8]. The unbridled expansion of the pandemic has generated extreme demands on health workers, causing physical exhaustion, psychological stress and contributing to mental illness such as Burnout syndrome [9]. The burden was further exacerbated by insufficient protective measures for the health workforce [10].

The actions developed by the National Health Surveillance Agency (ANVISA) were fundamental in reducing the spread of the virus in terms of epidemiological monitoring and preventative and coping recommendations [11]. Regarding the protection of health workers, ANVISA published, Technical Note No. 4 of 30 January 2020, recommending prevention and control measures to be adopted during assistance to suspected or confirmed cases of COVID-19 [12].

Given the proximity to patients and exposure to droplets and aerosols generated in procedures during care activities, oral health workers, the population covered by this study, showed a high risk of COVID-19 infection [13,14,15,16,17,18,19,20,21,22]. Analyzing data of confirmed cases of COVID-19 among dental professionals in relation to the numbers that affected the general population of the country, it was demonstrated a cumulative incidence of 5% higher among workers [23]. In order to reduce the transmission of COVID-19 through the generation of aerosols, elective dental procedures were suspended, and only emergency care was maintained [12]. 

In the face of early outbreaks of COVID-19, changes in dental practice have been investigated through population-based surveys worldwide [24,25,26,27,28,29,30]. In Brazil, studies have been conducted focusing on dental surgeons from restricted areas [31,32,33,34,35]. Thus, in an attempt to fill gaps and deepen the analysis, the present multicentric research was developed. This study aimed (1) to describe the surveillance and biosafety measures adopted by dentists, dental hygienists, and dental assistants who worked in the Southern Region of Brazil and (2) to evaluate access to information in the context of the COVID-19 pandemic.

## 2. Materials and Methods

### 2.1. Study Design and Population

This was a descriptive, cross-sectional, and multicenter study. It was conducted between August and October 2020 through the application of a virtual questionnaire targeted to dentists, dental hygienists and dental assistants who have worked in the three states of the Southern Region of Brazil (Paraná, Santa Catarina and Rio Grande do Sul). In April 2020, there were a total of 81,531 oral health workers in the Southern Region, according to data from the Federal Council of Dentistry. The final sample comprised 2560 participants. 

The employment status of the study participants was characterized as being from the public sector (SUS), from the private sector (private dental clinic), from university settings (dental clinic education), from the armed forces and from social promotion sectors of industrialists (SESI, SENAI and SESC). Detailed descriptions of the study design have been published previously [36]. 

The research project has been approved by the Research Ethics Committees of the university centers responsible for conducting the research in the states: State University of Ponta Grossa (CAAE n° 31720920.5.1001.0105; opinion 4.024.593), Federal University of Santa Catarina (CAAE: 31720920.5.2001.0121, opinion 4.226.476), Federal University of Parana (CAAE: 31720920.5.3001.0102, opinion 4.312.933) and Federal University of Rio Grande do Sul (CAAE: 31720920.5.2002.5530, opinion 4.071.063). All participants signed an informed consent form online.

### 2.2. Eligibility Criteria

The inclusion criteria for participants were: (a) being a dentist, technician, or dental assistant with active registration in the Regional Councils of Dentistry (CRO); (b) working in direct contact with patients; (c) being able to provide informed consent. The dentists, dental hygienists, or dental assistants who did not meet the above criteria were excluded from the study.

### 2.3. Pilot Study

The questionnaire was developed by the group of researchers involved in the present multicenter study and the validation of this instrument was performed in two stages previously described [36]. Firstly, eight experts with extensive experience in public health initially evaluated the instrument. The invitation to the reviewers was made via e-mail using a Google Forms^®^ form. In addition, they were asked to evaluate the degree of importance of each question. Secondly, the questionnaire was applied to 35 oral health workers (24 dentists, 6 dental hygienists, and 5 dental assistants) from different Brazilian states.

### 2.4. Data Collection

The final data collection instrument was composed of 47 questions, divided into three blocks: (a) sociodemographic profile of education and work; (b) health-care-related supplies and biosafety measures recommended by Technical Note GVIMS/GGTES/ANVISA no. 04/2020; (c) and professional practice and access to information. The questions were organized on a five-point Likert scale, for frequency type (1—never, 2—rarely, 3—sometimes, 4—most of the time, and 5—always) and agreement type (1—totally disagree, 2—partially disagree, 3—neither agree nor disagree, 4—agree, and 5—totally agree).

The questionnaire was made available online on Google Forms^®^ and, to cover the entire universe chosen for the study, the participation link was sent via e-mail by the CRO along with the consent form. After the first invitation, answers to the form were monitored and two more invitations were made in a 15-day interval. The time to fill out the instrument was approximately 20 min.

Data collection strategies were adopted for the dissemination of this study. During the data collection period, a broad dissemination strategy was consolidated by partnerships with the health authorities, educational institutions and professional category associations of each state, reports on social networks (WhatsApp^®^, Instagram^®^ and Facebook^®^) and dissemination events in Live Streaming on Youtube^®^.

### 2.5. Reproducibility

The reproducibility of the questionnaire was checked throughout data collection, and the participant was invited to answer the survey again after 7 to 10 days of the first response. Fifty e-mail addresses were randomly selected, and each selected participant received an individual code and the link to the form at the indicated address, with the purpose of making data parity possible. In case of no response within three days, an invitation was sent to the next individual on the list in his respective professional category.

The agreement obtained in the test–retest was from 84% to 100% for the categorical variables. The correlations for the questions with continuous and ordinal answers (on a Likert-type scale) showed an intraclass correlation coefficient (ICC) ranging from 0.4 to 1.0. The instrument showed adequate internal consistency, with a Cronbach’s alpha coefficient equal to 0.86.

### 2.6. Database and Data Analyzes

The database was reviewed, and duplicate records were removed. Duplicate responses were analyzed by initially checking for the presence of identical responses to the three open-ended questions. Once the total similarity was identified, the fields of personal characteristics and coded answers were checked, and the duplicate answers excluded.

The statistical analysis was performed using the *Stata* program. Continuous variables were summarized as mean (±standard deviation) and 95% confidence interval and categorical variables as absolute (n) and relative (%) frequencies (±standard deviations).

## 3. Results

The final sample was composed of 75.8% dentists, 15.7% dental assistants, and 8.5% dental hygienists, with the majority being female (78.4%). The predominant age groups were between 25 and 39 years (48.1%) and 40 and 59 years (43.2%). Regarding the area of residence, 44% of participants were living in the state of Paraná, 30.9% in Santa Catarina, and 25.1% in Rio Grande do Sul. 

Concerning dentists, 59.4% were specialists and of these, 19.9% were in public health. The type of employment was characterized by 52.7% in the public sector and 37.7% in private clinics. Approximately, 70% of oral health workers reported absence from work during the first wave of the pandemic of COVID-19. In relation to health aspects, 88.3% of professionals reported no increased risk factors or conditions for COVID-19 and 46.1% had not been tested for the disease (Table 1).

Of the surveillance measures adopted to control the spread of COVID-19 in health services, the preventive practices that obtained the highest mean scores were the investigation of respiratory infection symptoms when scheduling appointments (4.4 ± 1.1); the adoption of distancing in the waiting room (4. 4 ± 1.0); the immediate isolation of patients with symptoms of respiratory tract infection (4.2 ± 1.4); urgency based on pre-established clinical protocols (4.1 ± 1.2); the presence of visual alerts in health services (4.0 ± 1.4); and professionals’ guidance to patients about COVID-19 (4.0 ± 1.2). Lower mean scores were recorded in the practices considered innovative for the scope of dental care, such as direct action in fast-track procedures of COVID-19 (2.4 ± 1.6) and the use of tools for patient telemonitoring (2.8 ± 1.6) (Table 2).

The biosafety measures adopted by the oral health workers that obtained higher averages were those related to disinfection of the face shield (4.8 ± 0.7), the reuse of the N95/PFF2 mask (4.1 ± 1.4), the correct removal of personal protective equipment (PPE) (4.1 ± 1.3), and environment disinfection (4.0 ± 1.4). The lowest means were related to activities that promote minimizing the generation of aerosols and oral secretions such as the use of absolute dental isolation (2.4 ± 1.5) and avoiding intraoral radiographs (2.9 ± 1.4) (Table 3).

Table 4 presents how oral health workers obtained access to technical standards and recommendations on dental care during the COVID-19 pandemic. In general, oral health workers highlighted greater access to other non-governmental documents (78.0%). However, differences were found according to the professional categories analyzed. There was a higher proportion of dentists (61.3%) who accessed the information made available by the dentistry class entities compared to dental assistants (30.4%) and dental technicians (36.7%). In contrast, the categories of dental assistants and dental technicians reported having more access to recommendations offered by public health agencies such as municipal and state authorities (52.9% and 55.0%, respectively).

In Table 5, the results about the access to training to prevent the spread of SARS-CoV-2 showed that 54.4% of the participants strongly agreed with having received orientation and that 53.4% applied the knowledge acquired about measures adopted in dental care in the face of COVID-19. Meanwhile, when asked whether they consider themselves informed and safe to work in the pandemic, only 38.2% of participants strongly agreed and 34.9% reported feeling anxious or worried about working properly during the pandemic.

## 4. Discussion

This study described dimensions of the impact of the pandemic of COVID-19 on the workforce of dentists, dental assistants and technicians—professional categories often neglected yet which play an important role in the Brazilian health care system. Our findings show that changes in the health services landscape were highlighted by measures of surveillance as the investigation of respiratory infection symptoms when scheduling appointments and the adoption of distancing in the waiting room. Biosafety measures to avoid aerosols were less adhered to by the use of intraoral radiographs and the rubber dam, while the perceived feelings of stress and anxiety were related to the correct use of PPE at work. Higher access to information was observed through non-governmental documents.

The study population was predominantly female, young adults and up to ten years of professional training. Such findings were also identified in similar investigations in Brazil [32,34]. Women represent 70% of the health workforce in the country [7], an equivalent proportion of women who make up the health workforce in the Americas [37]. The female health workforce has played a key role in tackling the pandemic. The data of the present study also reflect a current scenario of the Brazilian dental reality in which there is an increase in young professionals due to the exponential increase in undergraduate courses in dentistry in the last decade [38].

The findings of our study revealed that approximately 70% of oral health workers absented themselves from work during the first wave of the pandemic. Similar rates were found in other countries. In Japan, the suspension of regular dental clinic activities impacted on the worsening oral health of individuals [39]. In Poland, for example, 71.2% of oral health workers voluntarily suspended their clinical practice during the first wave, induced by the need to mitigate the disease spread, insufficient pandemic coordination, PPE shortage, risk of contagion, and the uncertainties such as fear and anxiety regarding the pandemic [29]. In France, 77.7% of oral health workers stopped their activities in an attempt to decrease contamination [40]. It is also worth mentioning that in several countries, dentists were not quit their jobs but were allowed to practice only emergency/urgent procedures to limit the movement of people and enhance the psychological burden on health workers caused by COVID-19 [41,42].

In this study, tests to investigate SARS-CoV-2 infection were performed by 46.1% of the professionals. However, a similar investigation conducted in the twenty-six Brazilian states and the federal district found a percentage of only 8% of oral health workers tested [32]. In other countries, many oral health workers were not tested in the first wave. In Spain and France, only 3.7% and 4.8% were tested, respectively [28,40]. The insecurity in facing the pandemic with the low rates of testing exposes the scenario of work precariousness already present before the pandemic [43].

Our results show the adherence of oral health workers to the surveillance conducts and guidelines concerning COVID-19, more specifically the activities performed in the vicinity of the dental office. Surveillance practices are fundamental as a public health response to COVID-19, and dental settings are valuable sites for their development [44]. These results are consistent with studies indicating that dentists know the methods of investigation of patients with suspected COVID-19 [32,45] and inform the population about widespread problems of the disease [13,30], and are able to make them aware of the disease [30].

Innovative dental practices, such as the COVID-19 fast-track (fast-flow tool for triage and care of COVID-19 cases) and the use of teledentistry, had moderate uses among the oral health workers who participated in the present study. A study found that teledentistry was used during the pandemic as an innovative solution to provide continuity of dental practices, allowing greater visibility for dental professionals in several countries [46]. More restricted data on the use of teledentistry verified in the study are justified by digital, attitudinal [47] and technological barriers [47,48], which still persist in Brazil.

The pandemic scenario encouraged dental professionals in various parts of the world to seek knowledge about teledentistry and to make use of this resource [46,47], bringing positive impacts and acceptance among patients [48,49]. Patients’ acceptance was expanded with digital health and its enhanced surveillance capabilities, resulting in changes in the relationships between professionals and patients [50]. The experiences encountered in dentistry during the pandemic in Brazil, a country with extensive territory, demonstrated that teledentistry may in the future reduce geographical barriers and promote the the strengthening of health care in SUS.

The present study demonstrated a high rate of adherence of oral health workers to biosafety measures. Similarly to other studies, the N95/PFF2 mask showed good compliance [45,51], along with the face shield that started to be used in all appointments [52]. In addition, professional perception of inadequate use of PPE may be associated with symptoms of mental health disorder, post-traumatic stress, emotional suffering, and impacts on their general health [50]. The use and availability of PPE demonstrated a protective effect contributing to the confidence and safety of oral health workers in the continuity of dental care [53].

Low adoption of procedures to avoid aerosol generation was observed in the present study. Such results corroborate what was found in Germany where the main difficulties for this were insufficient knowledge, guidelines and recommendations, as well as the availability of equipment and high costs of PPE [54]. In Turkey, for instance, 90.6% of participants were concerned about aerosol-generating procedures; however, only 53.3% suspended these procedures [55].

Our study indicates significant rates of responses regarding access to unofficial documents which was observed in multinational research where the initial source of information had been the internet, followed by social media [30]. Oral health workers are engaged in the prevention and control of COVID-19 and need to be guaranteed access to reliable information [56]. High levels of knowledge, awareness and appropriate professional practices have been observed in other studies [13,55,57,58,59]. During the first wave in Turkey, 58.1% of oral health workers reported receiving training at their workplaces [57], while in Brazil, about 83% of dentists reported not having received specific training to control coronavirus transmission in the workplace [35].

The pandemic impacted the personal and professional lives of our study participants, with consequences on mental health, the high rates of anxiety reported. Anxiety and stress imposed on oral health workers increased as the pandemic spread globally, with even higher levels depending on the workplace. In the initial phase of the pandemic, 87% of oral health workers from 30 countries were afraid of becoming infected with COVID-19 at work. The SARS-CoV-2 virus infected 9.3% of the oral health workers evaluated worldwide before vaccination [60]. The number of infected individuals and mortality rates increased sharply in the same period [13]. In Poland, 56% of professionals were worried about the pandemic [59]. Uncertainty and fear about the future, together with social isolation, revealed the importance of making mental health care programs accessible to the entire population, including dentists, dental assistants, and dental technicians [43].

The present study has strengths and limitations. The pioneering character of this multicenter study using a sample of dentists, dental hygienists, and dental assistants during the first wave of the pandemic should be highlighted. However, care must be taken in the external validity of our results. First, our findings were determined within a political and social context. Second, our results may be related to the fact that the Southern Region of Brazil stands out for the best sociodemographic conditions, with a better ratio of health professionals and an organized public health network, factors that need to be considered in the extrapolation of data for the Brazilian context. Third, although there has been careful methodology, there is a possibility of social desirability bias as a result of the tendency of the participants to choose responses they believe are more socially desirable or acceptable rather than choosing responses that are reflective of their true thoughts or feelings.

## 5. Conclusions

This study has the potential to contribute to the restructuring of the dental clinic for the post-pandemic reality. Due to the national political scenario of ambiguities and tensions around the measures to be adopted for the control of the pandemic of COVID-19, the impaired consensus among the federated entities brought an unsafe scenario for the health workers in Brazil. However, our findings allow us to conclude that there was proper adherence to the guidelines on surveillance and biosafety indicated for the control of COVID-19. Furthermore, the adequate use of PPEs seems to be related to the self-perceived stress and anxiety of oral health workers, and the biosafety measures observed to be the most incorporated in dental practices were the use of N95/PFF2 masks and face shields.

The rate of work leave for dental professionals in Brazil was similar to that found in other countries, worsening the access to health care for the population with installed dental problems.

Finally, oral health workers showed a modest rate of participation in dental and interprofessional practices considered innovative in the attention to COVID-19, such as participation in the COVID-19 fast-track and/or the use of teledentistry. However, it is alarming that oral health workers’ access to information was in greater numbers through unofficial documents. Further research may be conducted investigating the access of oral health workers to scientific protocols in other contexts.

## Figures and Tables

**Table 1:** Sociodemographic, education, work, and health characteristics of the sample of oral health care workers from Brazil, August–October, 2020.

Variables	n	%	CI 95%
*Gender* (n = 2558)			
Female	2005	78.4	76.9–79.9
Male	553	21.6	20.1–23.1
*Age (years)* (n = 2560)			
18–24	134	5.2	4.4–6.1
25–39	1231	48.1	46.0–50.1
40–59	1105	43.2	41.3–45.2
≥60	90	3.5	2.8–4.2
*Residence (n = 2560)*			
Paraná	1127	44.0	42.0–46.0
Santa Catarina	790	30.9	29.1–32.7
Rio Grande do Sul	643	25.1	23.4–26.8
*Occupation (n = 2560)*			
Dentists	1941	75.8	74.2–77.4
Dental assistants	401	15.7	14.3–17.1
Dental technicians	218	8.5	7.5–9.6
*Completion of training (years)* (n = 2553)			
Up to 10	1135	44.5	42.5–46.4
11–20	695	27.2	25.4–29.0
≥20	723	28.3	26.6–30.1
Higher graduate level * (n = 1941)			
Specialization/Residency	1153	59.4	57.2–61.6
Master	256	13.2	11.7–14.7
PhD	172	8.9	7.6–10.1
None	360	18.5	17.0–20.2
*Postgraduate areas*^#^ (n = 1941)			
Public health	387	19.9	18.2–21.7
Clinical specialties ^#^	1188	61.2	59.0–63.4
None	360	18.5	16.7–20.4
Not informed	6	0.3	0.1–0.5
*Working sector (n = 2560)*			
Public ^¶^	1350	52.7	50.8–54.5
Private	966	37.7	36.0–39.6
Other	244	9.5	8.4–10.7
*Risk factors for severe forms of COVID-19* (n = 2558)			
Only age over 60 years old	62	2.4	1.8–3.0
Health condition only	219	8.6	7.6–9.6
Age over 60 and health condition	19	0.7	0.4–1.1
None	2258	88.3	87.1–89.4
*Absence from work during the pandemic* (n = 2560)			
No	769	30.0	28.2–32.1
Yes, due to suspected or confirmed COVID-19	336	13.1	11.9–14.5
Yes, due to other reasons	1455	56.8	54.7–58.8
*Testing for COVID-19* (n = 2560)			
No	1179	46.1	44.0–48.0
Yes	1381	53.9	52.0–56.0

* Only dentists included. ^#^ Most cited areas of dentistry: orthodontics, implantology, dental prosthesis, endodontics, periodontics, and pediatrics. ^¶^ Brazilian National Health System.

**Table 2:** Sample of oral health care workers’ distribution regarding the adoption of surveillance, planning and risk management measures to control the dissemination of COVID-19 in health services. Brazil, August–October, 2020.

Organization of Health Services (Surveillance, Planning and Management)	Always (Score 5) n (%)		Very Often (Score 4) n (%)		Sometimes (Score 3) n (%)		Rarely (Score 2) n (%)		Never (Score 1) n (%)		Mean *	(SD)
Suspended elective procedures and care restricted to urgency/emergency	961	(37.5)	702	(27.4)	460	(18.0)	242	(9.5)	166	(6.5)	3.8	(1.2)
Participation in decision making about changes in work during the pandemic	1321	(51.6)	557	(21.8)	245	(9.6)	121	(4.7)	173	(6.8)	4.1	(1.2)
Reduced workload or professional turnover to minimize the risk of contamination	916	(35.8)	259	(10.1)	354	(13.8)	220	(8.6)	784	(30.7)	3.1	(1.7)
Worked directly in COVID-19 reception/sorting/fast-track procedures	763	(29.8)	276	(10.8)	398	(15.6)	219	(8.6)	863	(33.7)	2.9	(1.7)
Investigation of respiratory infection symptoms in appointment scheduling	474	(18.5)	222	(8.7)	393	(15.4)	289	(11.3)	1144	(44.7)	2.4	(1.6)
Patients with symptoms of respiratory tract infection immediately isolated	1726	(67.4)	319	(12.5)	215	(8.4)	111	(4.3)	117	(4.6)	4.4	(1.1)
Waiting room respecting the minimum distance of 1 m between people	1533	(59.9)	319	(12.5)	120	(4.7)	100	(3.9)	280	(10.9)	4.2	(1.4)
Availability of visual alerts in the health service	1709	(66.8)	446	(17.4)	195	(7.6)	100	(3.9)	58	(2.3)	4.4	(1.0)
Urgency based on pre-established clinical protocols	1451	(56.8)	345	(13.5)	250	(9.8)	157	(6.1)	287	(11.2)	4.0	(1.4)
Orientation of patients about COVID-19	1296	(50.6)	463	(18.1)	415	(16.2)	185	(7.2)	156	(6.1)	4.0	(1.2)
Use of digital tools for teleorientation or telemonitoring	641	(25.0)	257	(10.0)	353	(13.8)	268	(10.5)	912	(35.6)	2.8	(1.6)
Interaction with other health professionals	849	(33.2)	550	(21.5)	683	(26.7)	248	(9.7)	214	(8.4)	3.6	(1.3)

* Excluded the answers ‘do not know’.

**Table 3:** Sample of oral health care workers’ distribution regarding the adoption of biosafety measures in health services. Brazil, August–October, 2020.

Work Biosafety	Always (Score 5)		Very Often (Score 4)		Sometimes (Score 3)		Rarely (Score 2)		Never (Score 1)			
	n	(%)	n	(%)	n	(%)	n	(%)	n	(%)	Mean *	(SD)
Disinfection of the environment by a trained professional with appropriate PPE	1386	(54.2)	403	(15.7)	226	(8.8)	185	(7.2)	307	(12.0)	4.0	(1.4)
Disinfection of suction hoses	1143	(44.7)	348	(13.6)	302	(11.8)	245	(9.6)	367	(14.3)	3.7	(1.5)
Use of sterile micromotors at every dental appointment	983	(38.4)	273	(10.7)	270	(10.6)	313	(12.2)	653	(25.5)	3.2	(1.7)
Intraoral radiographic examinations were avoided	372	(14.5)	604	(23.6)	589	(23.0)	332	(13.0)	573	(22.4)	2.9	(1.4)
Performing four-handed dental procedures	1077	(42.1)	387	(15.1)	307	(12.0)	328	(12.8)	400	(15.6)	3.6	(1.5)
Use of the dental dam in high-rotation procedures	315	(12.3)	297	(11.6)	406	(15.9)	316	(12.3)	1072	(41.9)	2.4	(1.5)
Procedures that generate aerosols were avoided	508	(19.9)	663	(25.9)	546	(21.3)	359	(14.0)	422	(16.5)	3.2	(1.4)
Use of suction system (vacuum pump)	1132	(44.2)	165	(6.4)	136	(5.3)	76	(3.0)	954	(37.3)	3.2	(1.9)
Proper removal of personal protective equipment	1362	(53.2)	546	(21.3)	241	(9.4)	141	(5.5)	216	(8.4)	4.1	(1.3)
N95/PFF2 mask reuse with proper criteria	1437	(56.2)	362	(14.1)	251	(9.8)	95	(3.7)	285	(11.1)	4.1	(1.4)
Disinfection of face shield	2220	(86.8)	150	(5.9)	55	(2.1)	26	(1.0)	54	(2.1)	4.8	(0.7)

* Excluded the answers ‘do not know’.

**Table 4:** Sample of oral health care workers’ distribution regarding the aspects related to access to technical standards and recommendations on dental care during COVID-19 pandemic. Brazil, August–October, 2020.

Variables	Dentists		Dental Assistants		Dental Hygienists		Total	
	n	(%)	n	(%)	n	(%)	n	(%)
*Access to technical standards and recommendations*	1132	(58.3)	170	(42.4)	104	(47.7)	1406	(54.9)
Technical note GVIMS/GGTES/ANVISA N° 04/2020	1189	(61.3)	122	(30.4)	80	(36.7)	1391	(54.3)
Recommendation booklet of the Federal Council of Dentistry (CFO)	1195	(61.6)	183	(45.6)	101	(46.3)	1479	(57.8)
Recommendation booklet of the Regional Council of Dentistry (CRO) from own state	310	(16.0)	22	(5.5)	12	(5.5)	344	(13.4)
Recommendation booklet of the Regional Council of Dentistry (CRO) from other state	1011	(52.1)	212	(52.9)	120	(55.0)	1343	(52.5)
Recommendations from the Municipal/State Secretariat	81	(4.2)	31	(7.7)	15	(6.9)	127	(5.0)
None	1455	(75.0)	357	(89.0)	184	(84.4)	1996	(78.0)
Other documents *	1132	(58.3)	170	(42.4)	104	(47.7)	1406	(54.9)

* Any source of information without identification of the agency responsible for the information accessed.

**Table 5:** Sample of oral health care workers’ distribution regarding training/education during COVID-19 pandemic. Brazil, August–October, 2020.

Training on COVID-19	Strongly Agree (Score 5)		Agree (Score 4)		Undecided (Score 3)		Disagree (Score 2)		Strongly Disagree (Score 1)			
	n	%	n	%	n	%	n	%	n	%	Mean *	(SD)
I consider that I received guidance at my workplace regarding measures to be taken during the COVID-19 pandemic	1392	(54.4)	683	(26.7)	164	(6.4)	145	(5.7)	148	(5.8)	4.1	(1.3)
I was able to apply the knowledge acquired in training/education about COVID-19 to modify my practice	1051	(53.4)	656	(33.3)	132	(6.7)	58	(2.9)	29	(1.4)	4.6	(1.1)
I feel sufficiently enlightened and secure to work properly in dental practice during the COVID-19 pandemic	978	(38.2)	1017	(39.7)	179	(7.0)	206	(8.0)	141	(5.5)	3.9	(1.2)
I feel anxious and concerned to work properly in dental practice during the COVID-19 pandemic	894	(34.9)	841	(32.9)	227	(8.9)	257	(10.0)	306	(12)	2.2	(1.3)

* Excluded the answers ‘do not know’.

## Data Availability

The data that support the findings of this study are available from the corresponding author, F.V.B., upon reasonable request.
